# An RVE-Based Study of the Effect of Martensite Banding on Damage Evolution in Dual Phase Steels

**DOI:** 10.3390/ma13071795

**Published:** 2020-04-10

**Authors:** Emin Erkan Aşık, Emin Semih Perdahcıoğlu, Ton van den Boogaard

**Affiliations:** Chair of Nonlinear Solid Mechanics, Faculty of Engineering Technology, University of Twente, 7500AE Enschede, The Netherlands; e.e.asik@utwente.nl (E.E.A.); a.h.vandenboogaard@utwente.nl (T.v.d.B.)

**Keywords:** steel, dual phase, void growth, crystal plasticity, strain gradient plasticity, polycrystalline material, martensite morphology

## Abstract

The intent of this work is to numerically investigate the effect of second phase morphology on damage evolution characteristics of dual-phase (DP) steels. A strain gradient enhanced crystal plasticity framework is used in order to capture the deformation heterogeneity caused by lattice orientations and microstructural size effects. The investigation is focused on two different martensite distributions (banded and random) that are relevant for industrial applications. The effects of martensite morphology are compared by artificially generated 2D plane strain microstructures with initial void content. The Representative volume elements (RVEs) are subjected to tensile deformation imposed by periodic boundary conditions. Evolution of voids are analyzed individually as well as a whole and characterized with respect to average axial strain. It is found that during stretching voids exhibit varying evolution characteristics due to generation of inhomogeneous strain fields within the structure. The behavior of individual voids shows that the stress-state surrounding the void is different from the imposed far field macroscopic stress-state. The voids at the ferrite martensite interface and in ferrite grains of the randomly distributed martensite grow more than in the banded structure. On the other hand, voids formed by martensite cracking growth shows an opposite trend.

## 1. Introduction

The increasing trend toward lighter structures has led the automotive industry to shift towards Advanced High Strength Steels that are generally multiphase steels with complex microstructural morphologies, which triggers research not only in production of these materials but also in development of numerical tools to predict and evaluate material performance. Microstructural features, e.g., distribution and amounts of phases, grain size and shape variations, of these steels affect the stress and strain fields within the structure during deformation. Ductile failure behavior is highly dependent on the local conditions of stress and strain. It is, therefore, necessary to understand interrelated physical mechanisms that cause ductile material failure in a voided polycrystalline structure subjected to inelastic deformations.

Ductile fracture of crystalline metals is generally a result of nucleation, growth and coalescence of small internal voids. Void nucleation usually occurs by decohesion or fracture of second phase particles [1,2,3]. Once nucleated, voids evolve with plastic deformation in a stable manner until they start interacting with each other, which later leads to coalescence and failure. The same story line holds for multi phase alloys. For example, experimental studies on dual phase (DP) steels show that void nucleation occurs by cracking of martensite, between ferrite and martensite or within ferrite grains. In [4] it is reported that interface decohesion and martensite cracking are observed and their proportion varies depending on the martensite content. Similarly in [5] it was found that martensite cracking and interface decohesion both occur in DP600 steels while martensite cracks appear earlier while having lower number of incidents. According to [6] while all three damage mechanisms are observed in the non-commercial DP steel that they studied, as grain size decreases the ductile mechanisms such as interface decohesion and ductile damage in ferrite, become more dominant. In [7] for the commercial DP600 they studied the main void formation was due to martensite cracking that started at the ferrite–martensite interface although some voids in ferrite were observed. It is concluded in [8] that for the DP800 they studied no definitive failure mechanism could be found meaning all of them were equally possible. Asik [9], who studied damage mechanisms for the specific DP steel that are used in this study, found that all damage mechanisms are equally likely to occur. A general overview of literature on this subject can be found in [10].

Modeling of void evolution in ductile metals and the response of mechanical properties to void content, size and shape have been described in the literature starting with the early works on isolated void analyses of McClinktock [11], Rice and Tracey [12] and the constitutive framework by Gurson [13]. Later, Koplik and Needleman [14] compared predictions based on Gurson’s model to unit cell analyses. In these early studies, matrix surrounding the void was assumed isotropic, although recently plastic anisotropy of the matrix has been taken into account in unit cell calculations by employing either a Hill-type yield criterion or crystal plasticity formulations [15,16,17,18,19,20,21,22].

Yerra et al. [19] did calculations on void growth in body centered cubic (BCC) crystal under constant stress triaxiality. They observed strong dependence of void growth rate to crystal orientation. Moreover, the study also showed that a higher stress triaxiality resulted in faster void growth. More recently, the study of Ling et al. [22] exhibited similar results for a face centered cubic crystal. In their work, unit cell simulations were compared with the porous single crystal model of Han et al. [18] for various grain orientations, stress triaxialities and initial void sizes. For the small voids at a stress triaxiality of 1, they have observed that the porous model underestimates the void growth for the [100] and [111] orientations while overestimates the void growth for the $\left[\bar{1}25\right]$ orientation. On the other hand, at a stress triaxiality of 3, both models predicted similar growth characteristics for the [100] and $\left[\bar{1}25\right]$ orientations, but overestimated the void growth for the [111] orientation. This was attributed to the distortion of the voids at that orientation. They have observed very similar results in the simulations with higher initial void fraction.

Shu et al. [21] investigated dependency of void size on void growth rate. They used a scale dependent elasto-viscoplastic framework for unit cell calculations and concluded that small voids had a tendency to grow slowly compared to large voids. Moreover, Borg et al. [23] and Tvergaard et al. [24] concluded that void growth rate for small voids was suppressed for low stress triaxialities and effect of orientation was more pronounced for small voids.

Void growth in metals has been studied at different length scales. At small scales, discrete dislocation dynamics (DDD) and molecular dynamics (MD) simulations are versatile tools to investigate the effect of lattice orientations and void size on determining the characteristics of porous single crystals. In the study of Segurado et al. [25], growth of voids in isolated face centered cubic (FCC) single crystals under uniaxial and biaxial loading was investigated via DDD simulations. It was found that void growth was more dependent on lattice orientations in uniaxial stress state than in biaxial loading. In the study of Tang et al. [26], through MD simulations of void growth and coalescence in magnesium single crystals, it was shown that the pattern of plastic deformation, which was strongly dependent on crystal orientations and specimens size, influenced void growth. Moreover, MD simulations in the study of Potirniche et al. [27], showed a higher increase of void fraction in smaller specimens, which was attributed to the development of larger local stresses.

Most of the previous work in literature for unit cell calculations use single crystals. However, in order to come up with a generalized understanding, representative features of an engineering material, e.g., phases, phase distributions, grain size, should be included in the model. The goal of this work is to clarify the effects of second phase distribution and grain orientations on the mechanics of void evolution in polycrystalline dual phase steel microstructures at mesoscopic scale (in the level of  0.1–10 m). A strain gradient enhanced rate independent crystal plasticity formulation is used in order to capture the size dependent evolution of inhomogeneous plastic deformation.

In this paper, void evolution in 2D polycrystalline Representative volume elements (RVEs) with initial void content is considered. The RVEs are generated with size and grains orientations so that the average stress and strain response is indifferent to the present grain orientations. RVEs with two constituent phases (ferrite and martensite) have been used to elucidate behavior of voids in a DP steel under tensile loading condition. Depending on the grade, martensite phase exhibits more than 4 times grater yield strength than that of ferrite, which is higher than the mechanical strength response difference that can be caused by grain orientation variations.

For the simulations, voids are introduced in the structure which are placed based on the most common active damage mechanisms [9,28]. The cylindrical voids were placed to ferrite–martensite boundaries and into ferrite grains but being closer to martensite. Moreover, fully close voids were introduced to the martensite islands in order to mimic the behavior of the voids formed by martensite cracking.

In order to emphasize the effect of second phase distribution, two different microstructural morphologies are used. In one case a banded distribution of martensite are employed and for the other case randomly distributed martensite islands in a ferrite matrix is used. The first structure resembles the banded martensite distribution of commercial DP600 steels, which has a minimum ultimate tensile strength of 590 MPa. The banded structure in DP steel sheets originates from the elemental segregation during solidification and subsequent rolling process [9,29]. On the other hand, the second distribution (random martensite distribution) is used to compare the evolution of different types of damage in order to clarify the effect of banding on damage evolution in DP structures.

Furthermore, the effect of grain orientations is studied by using three different sets of random orientations for each type of microstructure. The results are investigated in terms of area change of individual damage events—i.e., voids at grains, martensite–ferrite interfaces and voids formed from cracked martensite islands—as well as the total evolution of damaged area (area of the damage incidents).

The paper is organized as follows. Section 2 introduces the crystal plasticity and strain gradient theory used in this study. Section 3 describes the generated realistic microstructures with initial void content and the finite element model used in this study. Section 4 shows the results and discussions on the evolution of voids and effect of microstructural morphology and lattice orientations. Finally, Section 5 presents a summary and the main conclusions of this work.

Throughout the paper the following notations are used: 1st order tensors and vectors are shown in bold face and lowercase letters (a), 2nd order tensors and matrices in bold face and uppercase letters (A) and 4th order tensors with blackboard bold face and uppercase letters (A). The single contraction of tensors is represented by a dot ($A\;\cdot \;b=A_{ij}\;b_{j}$), double contraction by a colon ($A:B=A_{ij}\;B_{ij}$). The dyadic (tensor) product is represented with the ⊗ sign (C=a⊗b, $C_{ij}=a_{i}\;b_{j}$). The cross-product (×) of two vectors (a and b) is defined as $c_{i}=\epsilon_{ijk}a_{j}b_{k}$, where $\epsilon_{123}=\epsilon_{231}=\epsilon_{312}=1$, $\epsilon_{321}=\epsilon_{213}=\epsilon_{132}=-1$ and all other combinations equal to zero. The gradient operator in orthogonal basis is defined as $\nabla \left(\left\{•\right\}\right)=\frac{\partial (\{•\})}{\partial x_{i}}\;e_{i}$.

## 2. Constitutive Model

In this section, we aim to summarize the constitutive model used for damage evolution analysis. In the current work, we employ the strain gradient enhanced rate independent crystal plasticity framework developed by Perdahcıoğlu et al. [30] and we extend the model to incorporate lattice rotations. The framework was implemented by using user subroutines UMAT and USDFLD in finite element package Abaqus/Standard (version 2017). For the details concerning the implementation, readers are referred to Perdahcıoğlu et al. [30] and Soyarslan et al. [31], where algorithmic description of the framework, the backward Euler solution scheme and gradient computation method were discussed. The framework is capable of capturing strain gradient effects as a results of both structural and microstructural gradients. Especially, microstructural strain gradients play an important role when the plastic deformation is highly heterogeneous, which is the case for multi phase steels. The following section is subdivided into two parts: (i) crystal plasticity formulation, (ii) strain gradient enhancement.

### 2.1. Crystal Plasticity

#### 2.1.1. Kinematics

In the current model, it is assumed that crystallographic slip is the only deformation mechanism responsible for plastic deformation which happens on slip systems. Other mechanisms, such as twinning or transformation induced plasticity effects, are not considered since they are not observed for this specific material. The slip systems are defined by unit vectors of slip direction ($s_{0}^{\left(\alpha \right)}$) and slip plane normal ($m_{0}^{\left(\alpha \right)}$) of the slip system α. The total deformation (elastic and inelastic) can be expressed by using the total deformation gradient which for finite deformation, can be multiplicatively decomposed in the form

(1) $$F={\hat{F}}_{e}\;\cdot \;F_{i}$$

where $F_{i}$ describes the inelastic deformation caused by glide of dislocations and ${\hat{F}}_{e}$ describes elastic stretching and lattice rotation of the plastically deformed material point at an intermediate configuration (#). This decomposition implies that the elastic-plastic deformation process takes place in two stages. First, there is a plastic flow of material from the initial configuration to intermediate configuration and a subsequent stage of elastic deformation from intermediate configuration to final deformed configuration. The total velocity gradient L is calculated as

(2) $$L={\hat{L}}_{e}+{\hat{F}}_{e}\;\cdot \;L_{i}\;\cdot \;{\hat{F}}_{e}^{-1}={\hat{L}}_{e}+{\hat{L}}_{i}$$

The inelastic part of the total velocity gradient tensor is calculated by the sum of shear rates (${\dot{\gamma }}^{\left(\alpha \right)}$) of the slip systems as [32,33]

(3) $${\hat{L}}_{i}=\sum_{\alpha }{\dot{\gamma }}^{\left(\alpha \right)}s^{\left(\alpha \right)}⊗\;m^{\left(\alpha \right)}$$

where

(4) $$s^{\left(\alpha \right)}={\hat{F}}_{e}\;\cdot \;s_{0}^{\left(\alpha \right)},\;m^{\left(\alpha \right)}={\hat{F}}_{e}^{-T}\;\cdot \;m_{0}^{\left(\alpha \right)}$$

Furthermore using the definition of the velocity gradient in Equation (A2) one can obtain the rate of deformation by taking the symmetric part of L which can similarly be decomposed into elastic and inelastic parts:

(5) $$\begin{array}{cc} D & ={\hat{D}}_{e}+{\hat{D}}_{i}\\ W & ={\hat{W}}_{e}+{\hat{W}}_{i} \end{array}$$

The elastic part of the deformation rate is used in updating the stress while the inelastic part is determined using the found shear rates on active slip systems, as described in the following section. The inelastic part of the spin gives the possibility to update the elastic rotation that is needed to determine the lattice vectors in the deformed configuration.

The detailed equations that are necessary for the implementation of the model presented above can be found in Appendix A.

#### 2.1.2. Flow Rule

In the rate independent formulation, slip occurs only on the slip systems where the resolved shear stress ($\tau^{\left(\alpha \right)}$) of the slip system α is equal to its slip resistance ($\tau_{f}^{\left(\alpha \right)}$). Hence, we can define $\phi^{\left(\alpha \right)}$ for each slip system as:

(6) $$\phi^{\left(\alpha \right)}=\tau^{\left(\alpha \right)}-\tau_{f}^{\left(\alpha \right)}\leq 0$$

The systems at which the equality in Equation (6) holds are called the active slip systems for slip and the deformation is plastic. In this paper, we define the slip rates ${\dot{\gamma }}^{\left(\alpha \right)}$ for all systems to be positive which necessitates consideration of both positive and negative slip directions as slip systems [34].

The elastic behavior in the global frame is defined by the relation between lattice corotational rate of Cauchy stress tensor and rate of elastic deformation as:

(7) $$\overset{▿}{\sigma }=C^{e}:D^{e}$$

At each time increment, Abaqus/Standard rotates the stress tensor σ to the global reference frame in corotational formulation [35,36].

The resolved stress on the slip system α is calculated by the projection

(8) $$\tau^{\left(\alpha \right)}=\sigma :P_{\mathrm{tot}}^{\left(\alpha \right)}$$

where σ is the Cauchy stress tensor and $P_{\mathrm{tot}}^{\left(\alpha \right)}$ is called total Schmid tensor, defined as:

(9) $$P_{\mathrm{tot}}^{\left(\alpha \right)}=P_{s}^{\left(\alpha \right)}+P_{\mathrm{ns}}^{\left(\alpha \right)}$$

The $P_{s}^{\left(\alpha \right)}$ component of $P_{\mathrm{tot}}^{\left(\alpha \right)}$ geometrically projects the applied stress on the slip system with the slip direction $s^{\left(\alpha \right)}$ and slip plane normal $m^{\left(\alpha \right)}$.

(10) $$P_{s}^{\left(\alpha \right)}=s^{\left(\alpha \right)}⊗\;m^{\left(\alpha \right)}$$

$P_{\mathrm{ns}}^{\left(\alpha \right)}$ describes the non-Schmid effects that are responsible for the tensile compression asymmetry behavior of body centered cubic (BCC) crystals due to the core structure of screw dislocations and it is calculated as [37]:

(11) $$P_{\mathrm{ns}}^{\left(\alpha \right)}=a{}_{1}\;\left(s^{\left(\alpha \right)}⊗\;m_{l}^{\left(\alpha \right)}\right)+a{}_{2}\;\left(m^{\left(\alpha \right)}\times s^{\left(\alpha \right)}\right)⊗\;m_{l}^{\left(\alpha \right)}+a{}_{3}\;\left(m_{l}^{\left(\alpha \right)}\times \;s^{\left(\alpha \right)}\right)⊗\;m_{l}^{\left(\alpha \right)}$$

where a${}_{1}$, a${}_{2}$, a${}_{3}$ are temperature dependent material parameters.

In the literature, non-Schmid behavior of BCC structures has been investigated extensively by many researchers and the reader is referred to these works [37,38,39,40,41,42,43,44]. Here, we adopt the formulation developed by Gröger et al. [37] and employed by Koester et al. [45] and Cereceda et al. [41]. In this formulation, Equation (11), the vector $m_{l}^{\left(\alpha \right)}$ is the normal of *non-glide* plane which forms an angle of 30${}^{0}$ with the glide plane normal $m^{\left(\alpha \right)}$. List of the vectors $s^{\left(\alpha \right)}$, $m^{\left((\alpha \right)}$ and $m_{l}^{\left(\alpha \right)}$ can be found in the works of Gröger et al. [37] as well as Cereceda et al. [41]. Moreover, the material specific parameters are used as a${}_{1}^{298K}=0.030$, a${}_{2}^{298K}=0.173$, a${}_{3}^{298K}=0.300$ from the works of Patra et al. [38] and Mapar et al. [46].

In this work we consider both ferrite and martensite to have BCC structure with the same lattice parameters. This is assumed due to the low carbon content present in dual phase steels, see Table 2 for the chemical composition of a representative DP600 grade dual phase steel. The carbon content of martensite in this steel can be estimated to be roughly around 0.5 in weight percentage (based on 18% martensite fraction, and no C solid solution in ferrite). Although the real value depends on other factors such as other solute atoms, the aspect ratio, c/a, of the martensite lattice on carbon content can be estimated according to literature [47] to be smaller than 1.01.

#### 2.1.3. Hardening Rule

In the current work, the main mechanism for work hardening was considered to happen by impediment of dislocation motion by increase in the forest dislocation density. Therefore for each slip system, a Taylor type hardening law [48] with physically based interaction matrix was employed as in Equation (12):

(12) $$\tau_{f}^{\left(\alpha \right)}=\tau_{0}+\mu b\sqrt{\sum_{\beta }Q^{\left(\alpha \beta \right)}\rho^{\left(\beta \right)}}$$

where $\tau_{0}$ is the strain independent lattice friction, μ is the shear modulus, *b* is the Burgers vector length, $\rho^{\left(\beta \right)}$ is the total dislocation density of the slip system β and $Q^{\left(\alpha \beta \right)}$ is a physically based interaction matrix, which is generated by discrete dislocation dynamics simulations and takes into account the geometric relationship between the slip systems. Hence, it is defined by each crystal structure.

The coefficients of interaction matrix $Q^{\left(\alpha \beta \right)}$ characterizes the strengthening of slip system α due to increase of dislocation density on β. It is composed of six possible independent interactions of type: self, coplanar, collinear, orthogonal, glissile and sessile [41,49,50,51]. Table 1 shows coefficients of the interaction matrix determined by Queyreau et al. [51] via discrete dislocation dynamics simulations for a BCC structure.

By following the arguments of Ashby [52], the total dislocation density $\rho^{\left(\alpha \right)}$ of a slip system α was considered to be the sum of statistically stored dislocation $\rho_{\mathrm{SSD}}^{\left(\alpha \right)}$ and geometrically necessary dislocation $\rho_{\mathrm{GND}}^{\left(\alpha \right)}$ densities.

(13) $$\rho^{\left(\alpha \right)}=\rho_{\mathrm{SSD}}^{\left(\alpha \right)}+\rho_{\mathrm{GND}}^{\left(\alpha \right)}$$

The evolution of $\rho_{\mathrm{SSD}}^{\left(\alpha \right)}$ is governed by shear rate of slip system α whereas $\rho_{\mathrm{GND}}^{\left(\alpha \right)}$ evolves by the gradient of the shear rate and it will be discussed in the next section. As in the work of Perdahcıoğlu et al. [30], the evolution of $\rho_{\mathrm{SSD}}^{\left(\alpha \right)}$ is based on a phenomenological constitutive law based on the linear ordinary differential Equation (14)

(14) $${\dot{\rho }}_{\mathrm{SSD}}^{\left(\alpha \right)}=\frac{{\dot{\gamma }}_{\left(\alpha \right)}}{\gamma^{\infty }}\left[\rho_{\mathrm{SSD}}^{\infty }-\rho_{\mathrm{SSD}}^{\left(\alpha \right)}\right]$$

where the terms $\rho_{\mathrm{SSD}}^{\infty }$ and $\gamma^{\infty }$ are constants that control the values for saturation of statistically stored dislocation density and the rate of saturation which are phenomenological descriptions of the balance between rate of dislocation production and annihilation [53].

### 2.2. Geometrically Necessary Dislocations (GND) Density: Strain Gradient Enhancement

This section gives the formulation that is required to calculate the evolution of $\rho_{\mathrm{GND}}^{\left(\alpha \right)}$ based on the gradient of slip rates. For calculating the evolution of geometrically necessary dislocation densities, we follow the formulations of Gurtin et al. [54] and Cermelli et al. [55] and the details of the implementation are given in Appendix B.

Once the shear rates on each slip system are known their gradient can be used to determine the Burgers tensor explicitly as

(15) $$G=F_{i}\;\cdot \;\mathrm{Curl}\;F_{i}$$

which involves the rate of screw (⊙) and edge (⊢) geometrically necessary dislocation densities on slip system α in the following form [54]:    

(16) $$\begin{array}{c} {\dot{\rho }}_{⊙,\mathrm{GND}}^{\left(\alpha \right)}=l_{0}^{\left(\alpha \right)}\;\cdot \;\nabla_{0}{\dot{\gamma }}^{\left(\alpha \right)} \end{array}$$

(17) $$\begin{array}{c} {\dot{\rho }}_{⊢,\mathrm{GND}}^{\left(\alpha \right)}=-s_{0}^{\left(\alpha \right)}\;\cdot \;\nabla_{0}{\dot{\gamma }}^{\left(\alpha \right)} \end{array}$$

where $l_{0}^{\left(\alpha \right)}$ is a lattice vector given as $l_{0}^{\left(\alpha \right)}=m_{0}^{\left(\alpha \right)}\times s_{0}^{\left(\alpha \right)}$.

The edge and screw GND densities represent the vector components of the total GND density and due to the definition of G, the unit of densities is not per area but rather per length and it is more a geometrical measure without any material specific input. It is therefore necessary to convert to per area by dividing by the length of the Burgers vector of the material. This yields the total GND density to be used in Equation (13) as

(18) $$\rho_{\mathrm{GND}}^{\left(\alpha \right)}=\frac{1}{b}\sqrt{{\left[\rho_{⊢,\mathrm{GND}}^{\left(\alpha \right)}\right]}^{2}+{\left[\rho_{⊙,\mathrm{GND}}^{\left(\alpha \right)}\right]}^{2}}$$

Computation of strain gradients is realized explicitly making use of a discrete gradient computation method proposed by Liszka and Orkisz [56]. An irregular grid of data points can be used with this method for the evaluation of the gradients. This method is used to approximate the unknown gradient vector by using a weighted least squares approach. To remedy the over-determinacy of the system of equations associated with the condition where the number of equations exceeds the number of unknowns we use the following sum of squares form

(19) $$f\left(\Upsilon \right)=\sum_{k=1}^{n}{\left[\frac{{\dot{\gamma }}^{\left(\alpha \right)}\left(r_{0}\right)-{\dot{\gamma }}^{\left(\alpha \right)}\left(r_{k}\right)+\Upsilon \;\cdot \;\Delta r_{k}}{\Delta r_{k}^{3}}\right]}^{2}\;,$$

where $1/\Delta r_{k}^{3}$ is the weighting factor. Minimization of f(Υ) with ∂f/∂Υ=0 gives the desired gradients. This procedure is implemented as a USDFLD subroutine for Abaqus.

In the case of polycrystal simulations, the gradient computation is limited within each domain of elements belonging to individual material definitions. This implies that the jump of the plastic strain across the grain boundaries is not treated as a source of GNDs.

## 3. Microstructure

In this section, 2D polycrystalline RVEs consisting of two phases (ferrite and martensite) have been used to investigate the evolution of voids in a DP steel under tensile loading condition. Fully closed voids were introduced inside martensite islands, cutting through the complete island. Moreover, two types of cylindrical voids were placed at two different location types. The first type of location was the interphase boundaries between ferrite and martensite. The second type was inside the ferrite grains with a martensite neighbor and towards the boundary between ferrite and the neighboring martensite island. The choice of these locations was based on the experimental observations, which show that in DP steels there is more than one active damage mechanism [9,28]. By adding the most common damage mechanisms into the model we aim to clarify and give an explanation on how one of the mechanisms becomes dominant over the others and on the effect of martensite banding on the evolution of these damage mechanisms.

Two different martensite morphologies were considered, namely banded and randomly distributed structures. Throughout the section, we have used the word *morphology* to specifically mention distribution of martensite islands in the ferrite matrix. For readability we have not used the word *distribution* every time but it has always been implied unless mentioned otherwise. The RVE with banded morphology was generated by considering a commercial DP600 steel (Tata Steel, Ijmuiden, The Netherlands). The same batch of this steel has been investigated experimentally before for damage mechanisms [9] and has the composition given in Table 2.

**Figure 1:**
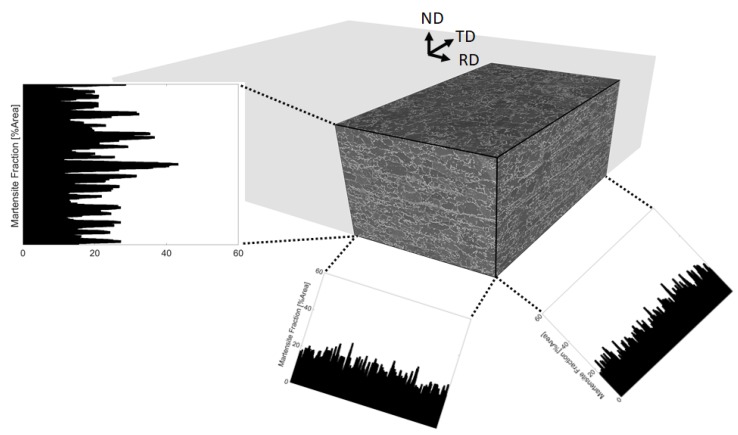
Typical microstructure of a commercial DP600 steel and distribution of vol% martensite where ND (normal direction) represents the direction normal to the plane of sheet and RD and TD represent the rolling and transverse directions of the sheet.

**Figure 2:**
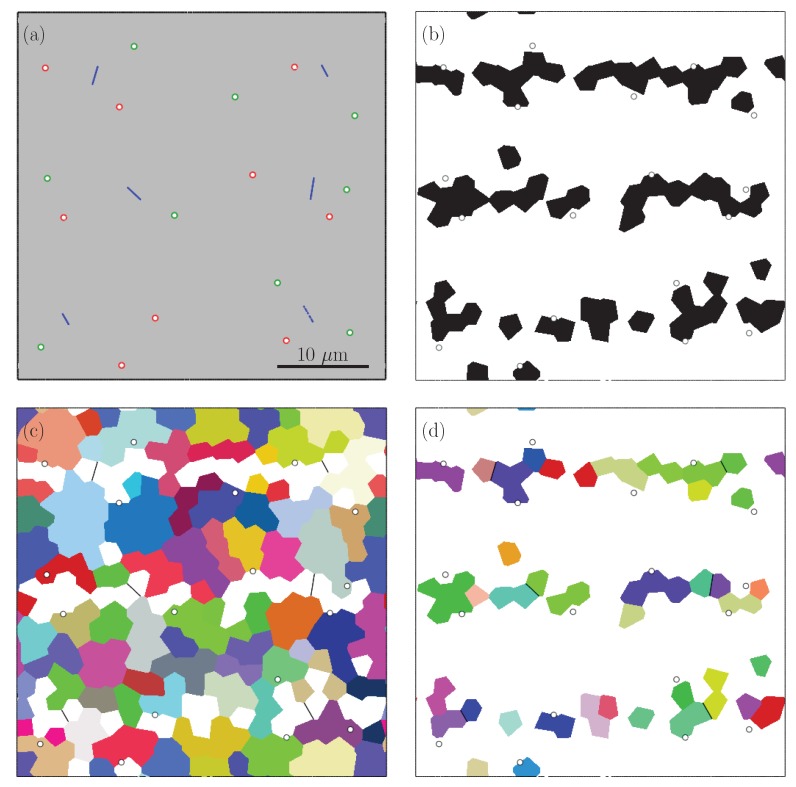
RVE with banded morphology, (**a**) voids (green: voids in ferrite, red: voids at the ferrite–martensite boundary) and cracks (blue), (**b**) ferrite (white) and martensite (black), (**c**) colors indicate 69 ferrite orientations, (**d**) colors indicate 29 martensite orientations.

**Figure 3:**
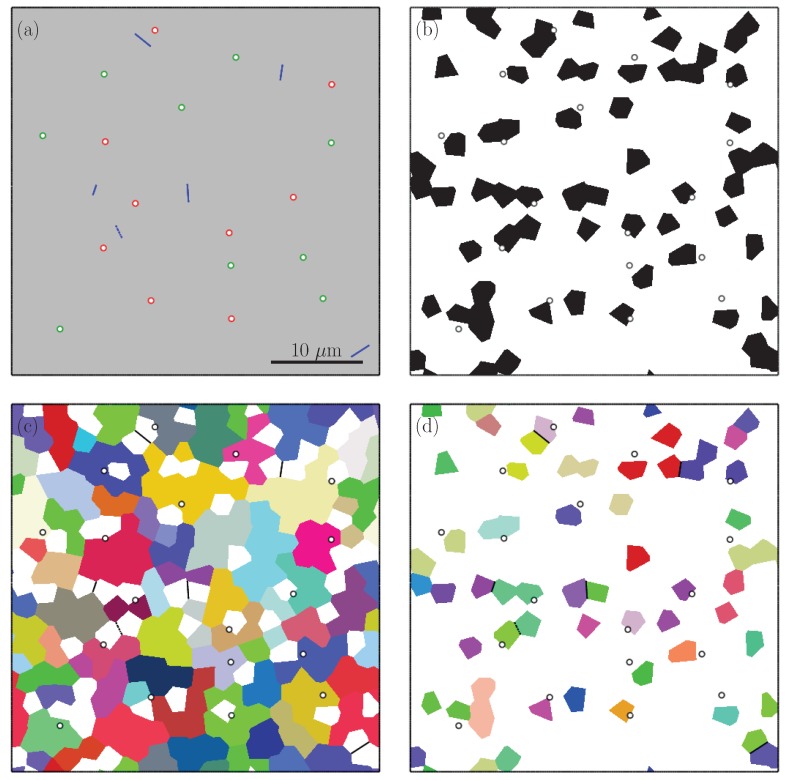
RVE with random morphology, (**a**) voids (green: voids in ferrite, red: voids at the ferrite–martensite boundary) and cracks (blue), (**b**) ferrite (white) and martensite (black), (**c**) colors indicate 67 ferrite orientations, (**d**) colors indicate 32 martensite orientations.

Figure 1 illustrates secondary electron (SE) images obtained from different cross-sections of the steel and the measured volume percentages of martensite distribution along rolling, transverse and normal directions. In [9] the experimental procedures for obtaining the images as well as the quantitative analysis methods are introduced. Using these methods, the martensite content was calculated by image analysis and it was found to be an average of 17.9±0.4 vol% martensite. From the martensite distribution charts, along the thickness direction a non-homogeneous, banded distribution of martensite can be seen. The spacing between two martensite bands were measured to be roughly 10 to 15 μm and in between the martensite bands there were 2–3 ferrite grains [9]. Moreover, grain analysis by electron backscattered diffraction revealed an average ferrite grain area of 22.5 μm${}^{2}$ and a martensite grain area of 5.6 μm${}^{2}$. According to these measurements an RVE with a size of 40×40 μm and containing 18.14 vol% martensite was generated, as shown in Figure 2, to represent a banded DP600 steel. For representing the RVE with random martensite morphology, grain sizes were generated with the same parameters and this yielded an RVE with a martensite content of 17.85 vol%, see Figure 3. The martensite morphologies of the generated RVEs can be seen in Figure 2b and Figure 3b, where black color represents the martensite phase. Moreover, Figure 2c,d and Figure 3c,d show the ferrite and martensite grains.

In the EBSD analysis no specific texture associated with each phase was observed and accordingly the tensile tests in different directions did not reveal any plastic anisotropy [9]. Therefore, orientations of the grains in both RVEs were assumed to be random and orientation variations within a martensite island were not taken into account. For each RVE, 3 randomly generated orientations sets were used in order to statistically compare damage evolution in the structure.

The RVEs were generated by using Microstructure Design Tool: Multilevel Voronoi (MLV) tessellation software developed by Tata Steel. The Multilevel Voronoi technique enables generating very complex grain structures and phase distributions compared to the standard Voronoi tessellation, which results in convex shaped grains. The basic principle of the technique is based on regrouping fine Voronoi structures by using a coarser tessellation depending on location of the seed points of fine Voronoi cells. The reader may refer to the works of Kok et al. [57] and Yadegari et al. [58] for more detailed explanations on the multilevel Voronoi approach, as well as the possibilities it presents in microstructure generation. The MLV software generates output files, that contain geometrical and orientation information to create microstructures. These files were used to create grains with defined orientations as parts in Abaqus/CAE by using the scripting language Python.

After RVEs were generated, different types of voids were introduced to the structure in the graphical user interface of Abaqus/CAE and the resulting RVEs were meshed with quadratic triangular plane strain elements (CPE6M). The boundary of voids and cracks were meshed finer for better discretization. Both RVEs contain 9 voids at the ferrite martensite interface and 9 voids inside ferrite grains with a radius of 0.3 μm and 6 completely cracked martensite grains shown in Figure 2a and Figure 3a, which makes an initial void percentage (%$f_{0}$) of 0.32. The initial void size was selected in a way to represent voids which are nucleated but not grown much. The RVEs were subjected to 15% tensile elongation by applying periodic boundary conditions which were imposed by tie constraints to opposing edges. Moreover, periodicity of the grains, which are located at the opposite edges of the RVEs, were ensured during the RVE generation procedure such that a grain at the right or top edge continues to the left or bottom edge.

### Parameter Identification

**Figure 4:**
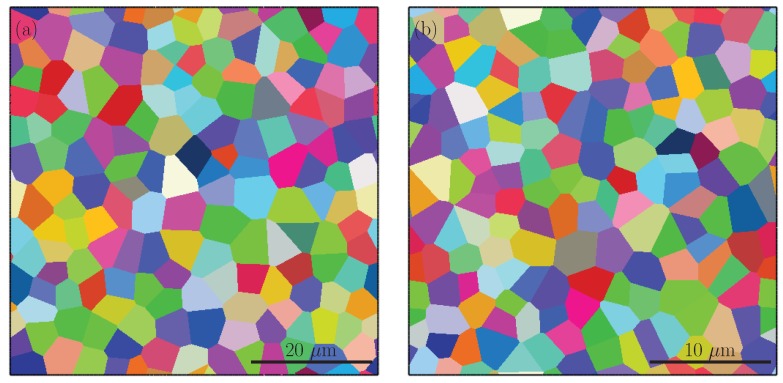
RVEs used to model (**a**) ferrite, (**b**) martensite. Colors represent the Voronoi cells with different grain orientations.

Since the aim of the simulations was to investigate the effect of martensite distribution on damage evolution in DP600 steels, the material properties of the phases were fitted to macroscopic stress–strain response of ferrite and martensite that was used by Ramazani et al. [59] in modeling dual phase steels. Parameter identification was done by employing an RVE consisting of either ferrite or martensite as represented in Figure 4.

For each phase, a Voronoi-based microstructure with 160 grains was generated and the RVEs were subjected to 7.5% tensile tensile stretch, while periodic boundary conditions were imposed. The stretching direction is the one horizontal to the image and parallel to the band orientation since the vertical direction is assumed to represent the thickness of the sheet. Considering that the constitutive model used in this study is size dependent the grain sizes of the single phase RVEs were generated to have similar grain sizes of constituent phases as the RVEs with dual phase structures and the simulations were repeated with 3 different sets of random orientations to check the representativeness. Moreover, in the current fitting the initial dislocation densities for each slip system of each phase were calculated from the total dislocation density that is reported in literature as $9.0\times 10^{7}$ mm${}^{-2}$ for ferrite [60] and $1.6\times 10^{9}$ mm${}^{-2}$ for martensite [61]. These densities were assumed to be equally distributed to the 12 possible slip systems yielding dislocation densities of $7.50\times 10^{6}$ mm${}^{-2}$ and $1.34\times 10^{8}$ mm${}^{-2}$ for each slip system of ferrite and martensite, respectively. The other parameters ($\tau_{0}$, $\rho^{\infty }$ and $\gamma^{\infty }$) given in Table 3 were used as fitting parameters.

In the used model, in total each phase has 7 parameters 2 of which are elastic and are determined by direct testing. One is the Burgers vector that depends on the lattice parameters of each phase which are known in literature for the ferrite and martensite in DP steels. Another one, i.e., the initial dislocation density, is taken from measurements reported in the literature for each phase. This leaves three parameters per phase, i.e., lattice friction, saturation dislocation density and saturation shear, to be fitted using a macroscopic tensile test. Lattice friction has a direct relation to the initial flow stress of the phase and the other two parameters influence the hardening behavior at large strains. Using these observations and the data found in [59] a calibration was performed to have a good correspondence with the macroscopic tensile test result.

**Figure 5:**
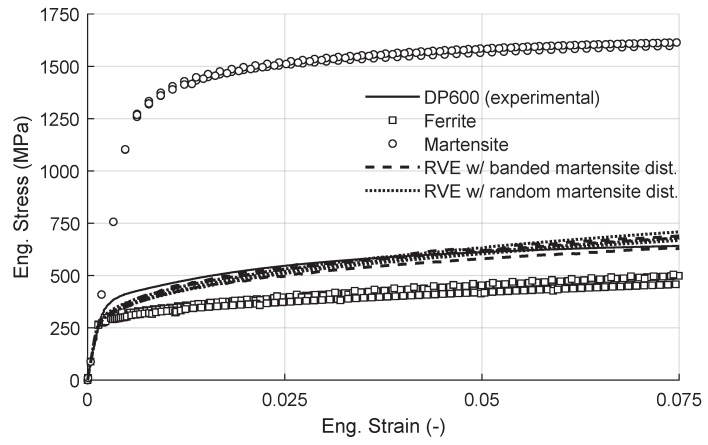
Stress–strain response of DP600 (experimental), individual phases and the different morphologies.

Mechanical response of RVEs with only ferrite or martensite is shown in Figure 5 with markers. Additionally, the figure also shows stress–strain curves of RVEs with banded and random morphologies and an experimentally measured DP600 curve. It can be seen that the obtained stress–strain curves for each phase are within the range that is reported in literature and the overall response of the RVE is in good correspondence with the experiment. With the current RVE size, we see that the stress response of DP600 structures shows a maximum scatter of 3%, which is sufficiently low for this study. However, if necessary, this scatter can be reduced by using larger sized RVEs at the cost of a higher solution time.

## 4. Results and Discussion

### 4.1. Evolution of Dislocation Densities

**Figure 6:**
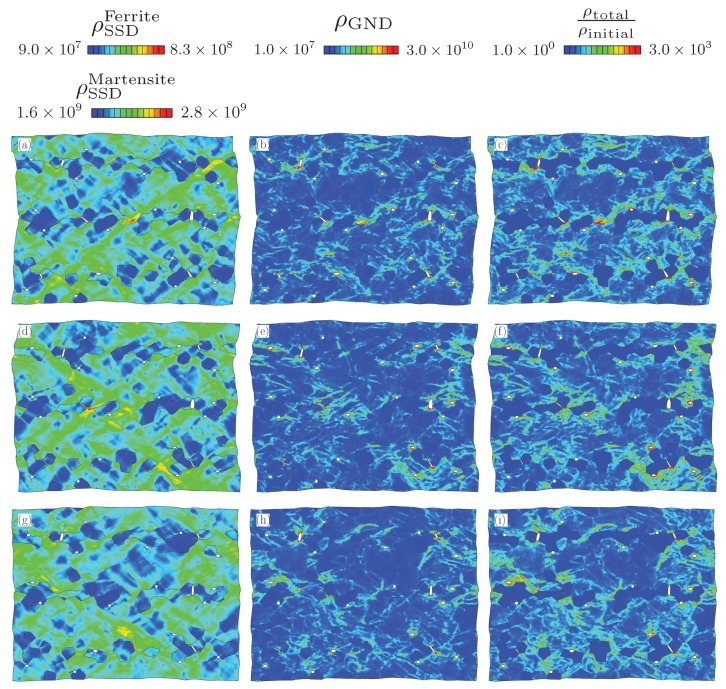
Distribution of (**a**,**d**,**g**) $\rho_{\mathrm{SSD}}$ (mm${}^{-2}$), (**b**,**e**,**h**) $\rho_{\mathrm{GND}}$ (mm${}^{-2}$), (**c**,**f**,**i**) Normalized dislocation density for banded morphology. Three rows represent 3 different orientation sets.

**Figure 7:**
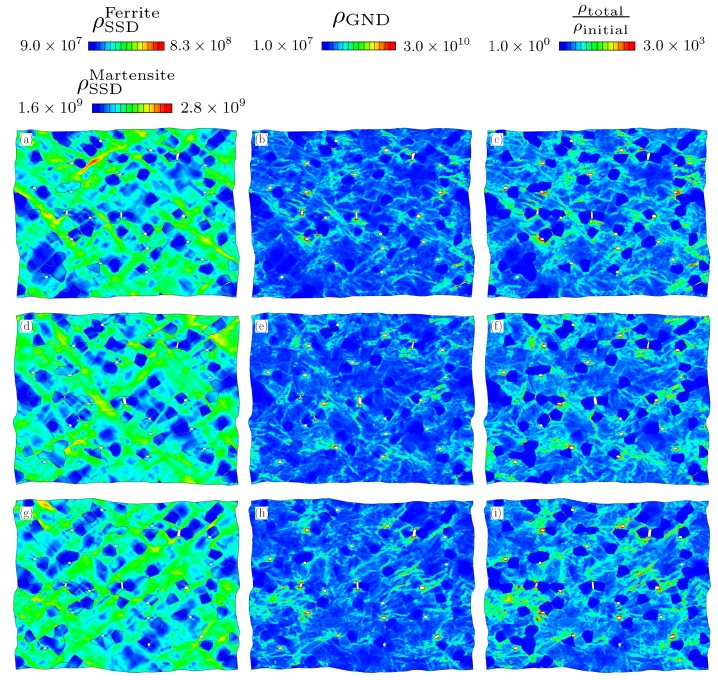
Distribution of (**a**,**d**,**g**) $\rho_{\mathrm{SSD}}$ (mm${}^{-2}$), (**b**,**e**,**h**) $\rho_{\mathrm{GND}}$ (mm${}^{-2}$), (**c**,**f**,**i**) Normalized dislocation density for random morphology. Three rows represent 3 different orientation sets

Here we compare the distribution of dislocation densities after deformation. This is done by first visualizing the deformed RVEs using plots of SSD and GNDs showing their spatial distribution. Two different scales were used for Figure 6a,d,g and Figure 7a,d,g due to the large difference between the initial statistically stored dislocation (SSD) density in ferrite and martensite. For both morphologies, deformation is not uniform but it concentrates in certain regions and forms shear bands. The shear bands can be identified from the distribution of SSD density, which accommodates and evolves with plastic strain. The main difference in the SSD density of two morphologies is the distribution of these bands. In the random morphology, shear bands form a finer pattern compared to that in the banded one. Based on this observation the following can be deduced. Ferrite in between the martensite islands has to deform to accommodate the prescribed deformation since the mechanical strength contrast between the two phases is large so that ferrite phase in any orientation is softer than martensite. The presence of a higher number of ferrite channels between the martensite islands in the random morphology yields finer shear band structure. However, the long and continuous martensite bands restrict the plastic flow causing coarser shear bands.

**Figure 8:**
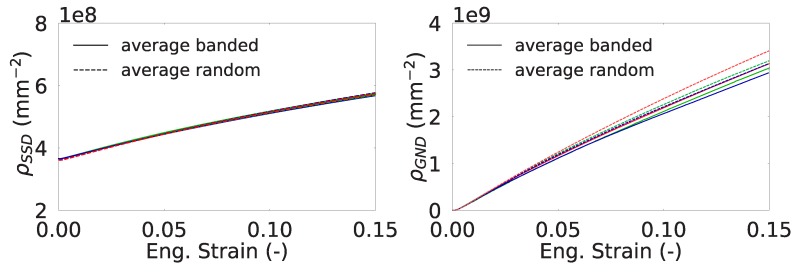
Averaged statistically stored dislocation (SSD) (**left**) and geometrically necessary dislocations (GND) (**right**) densities within the RVEs. Each color represents an orientation set.

**Figure 9:**
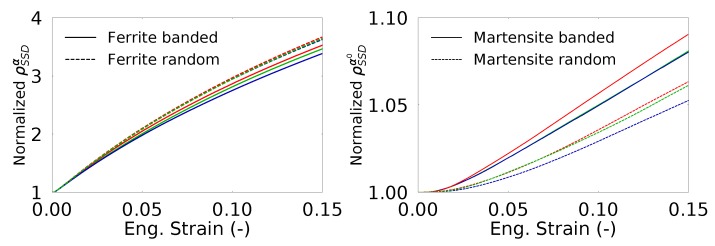
Evolution of normalized SSD density (with respect to $\rho_{\mathrm{SSD}}^{0}$) in the RVEs for ferrite (**left**) and martensite (**right**). Each color represents an orientation set.

The effect of martensite distribution and shear band formation can also be evaluated from the evolution of average SSD and GND densities in different phases. Figure 8 shows that the average SSD evolution is almost similar for all the cases where there is formation of higher GND density for the RVEs with random morphology. Figure 9 shows the change in SSD density in ferrite and martensite. The higher average SSD density of ferrite in the random morphology suggests accommodation of a higher amount of plastic strain than the ferrite phase in the banded morphology. In the meantime, martensite phase strains less and yields a lower amount of SSD density. From a stress point of view, this means that the continuous martensite bands carry more load compared the martensite islands in the random morphology. The scatter with respect to the average value due to different orientation sets can also be investigated. It is seen that the average normalized SSD density in ferrite of banded morphology has a scatter of 6.0% at 0.15 longitudinal strain, whereas that of random morphology is 1.7%, which means orientations in a banded structure play a more important role than a random morphology. On the other hand, scatter in average SSD density in martensite for both morphologies is much smaller at a value of 0.5%.

**Figure 10:**
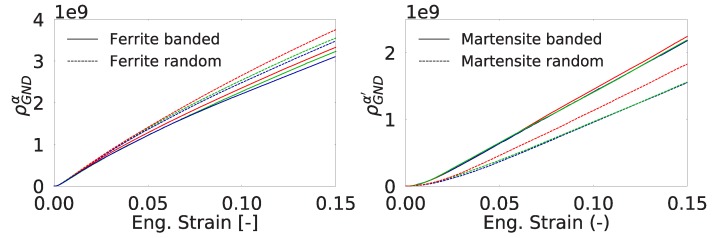
Evolution of GND density in the RVEs for ferrite (**left**) and martensite (**right**). Each color represents an orientation set.

The GND density distributions can be compared by using Figure 6b,e,h and Figure 7b,e,h. All the figures show clear localization of GND density around voids and at the tips of cracks. In addition, moderate GND densities ($5\times 10^{8}$ ${\mathrm{mm}}^{-2}$) in ferrite are present at the grain boundaries. In Figure 10 the total GND densities per phase within the random and banded RVEs are plotted. It is seen that the average GND density of ferrite evolves faster in the random morphology. This can be related to the finer structure of shear bands and higher SSD ($\rho_{\mathrm{SSD}}^{\alpha }$) content of random morphology. First, through the width of a shear band, there exists a gradient of slip resulting in GND accumulation. Since there are more shear bands in random morphology, they cause higher amount of GNDs. Secondly, ferrite deforms more in random morphology as indicated by the higher SSD ($\rho_{\mathrm{SSD}}^{\alpha }$) content. This increases the heterogeneity of plastic deformation and the GND content in ferrite. This trend is also observed in the average GND content of martensite. The higher amount of plastic deformation in martensite phase in the banded structure than the martensite islands of random morphology causes development of higher strain gradients. This is due to inhomogeneous distribution of plastic deformation within the martensite.

Figure 6c,f,i and Figure 7c,f,i show the distribution of normalized total dislocation densities with respect to the initial dislocation density ($\rho_{\mathrm{SSD}}$) of the structure. After normalizing the additional intensity of dislocation density due to the strength contrast between phases and the high density caused by presence of voids and cracks become clearly visible. It is seen between the figures that the dislocation density varies with different orientation sets, even if the average stress–strain curves are within a range of 3%. For example, the top right corner of Figure 6c has less dislocations than Figure 6f. However, there are also similarities. In all figures there are large regions of ferrite which are deformed less than the average of the structure. Moreover, around the damage occurrences, the dislocation density is always higher than in the rest of structure due to excess plastic deformation caused by stress concentration.

### 4.2. Evolution of Voids

**Figure 11:**
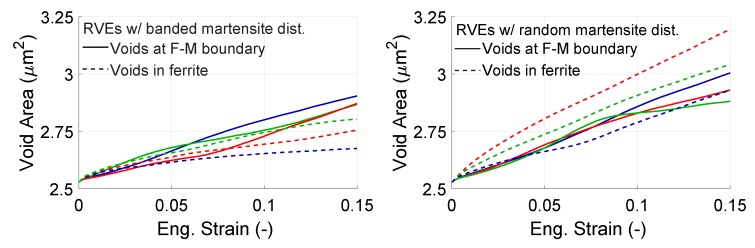
Evolution of total area of voids (**left**) banded, (**right**) random morphology for 3 orientation sets with total strain. Each color represents an orientation set.

This section elaborates on the evolution of voids found in the RVEs in terms of total area of the voids as well as the individual area of each void. Figure 11 shows the increase in the total void area for 2 morphologies and 3 sets of orientations each. The voids are separated into 2 different classes namely the voids at the interface (solid lines) and the voids in ferrite grains (dashed lines).

From Figure 11, we see the final average void area is bigger in the random morphology with an increase of 19.8%, whereas it is 12.4% in the banded morphology. If we compare the different types of voids (in-grain and at the interface), we see that the average void growth of in-grain voids were highly affected by the martensite morphology, whereas interface voids seem to be less effected. At the end of deformation, the average area of all in-grain voids increases 20.7% from 2.53 μm${}^{2}$ to 3.05 μm${}^{2}$ in random morphology. For the banded morphology, the growth of the same type of voids is at 8.5% corresponding to a total area of 2.74 μm${}^{2}$. However, the average growth of interface voids for both morphologies deviate less from each other. It is slightly larger for random morphology at 16.1% and 13.8% for the banded morphology. We can relate the higher percentage of in-grain void growth to the higher deformation of ferrite in random morphology, which we can see from the higher SSD density value in ferrite and lower SSD density in martensite, Figure 9. The higher SSD density in ferrite in random structures suggests two things. First, it means that the ferrite is plastically deformed more for that structure. Secondly, it means that the ferrite has hardened more. Since on the RVE deformation is prescribed, the voids have to grow more. In other words, the mechanical contrast between the void and the ferrite increases as ferrite hardens which forces the void to take more part in the deformation process. The storyline for interface voids is similar but this time the deformation of martensite is also crucial. It seems that since martensite does not deform as much as ferrite it stabilizes the void, the growth of interface voids depends not only on deformation of ferrite but also on the deformation of martensite.

**Figure 12:**
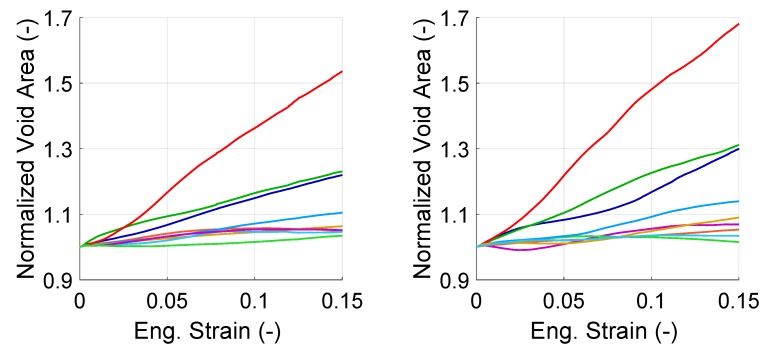
Evolution of the normalized area (with respect to initial area) of individual interface voids in banded (**left**) and random (**right**) morphology with total strain. Each color represents one void.

**Figure 13:**
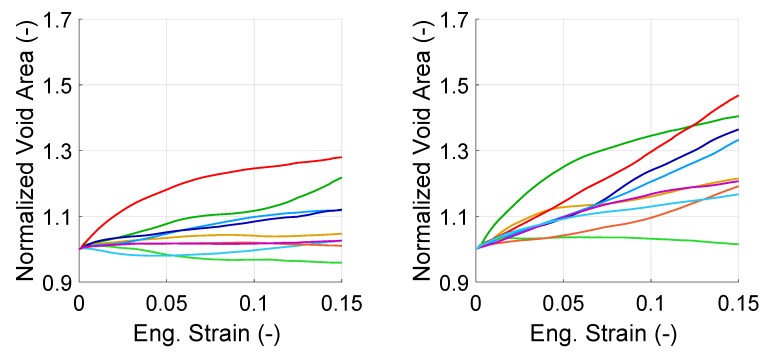
Evolution of the normalized area of individual in-grain voids in banded (**left**) and random (**right**) morphology. Each color represents one void.

Figure 12 and Figure 13 show the change of normalized void area for individual voids for only one set of orientations per structure. The first observation on the curves of the figures is that all voids evolved differently from each other. The varying behavior of the voids can be attributed to (i) the orientation of the grains with respect to the loading axis, (ii) distribution of plastic strain within the RVE. Secondly, for both void groups, there was at least one void which did not grow in size but shrunk during the deformation. Shrinkage of voids strongly suggests that there was either shear or compressive stress state surrounding those voids. Moreover, some of the voids show an initially growing trend which during the deformation process turns into shrinkage or vice versa, which can be attributed to change of local stress state. This is an important result since it shows the difference in evolution of the local stress state compared to the evolution of macroscopically applied stress state. Thirdly, in both structures, there was at least an in-grain void which grows 50%, which is 4–7 times more than the other voids.

### 4.3. Evolution of Voids Formed by Cracking of Martensite

**Figure 14:**
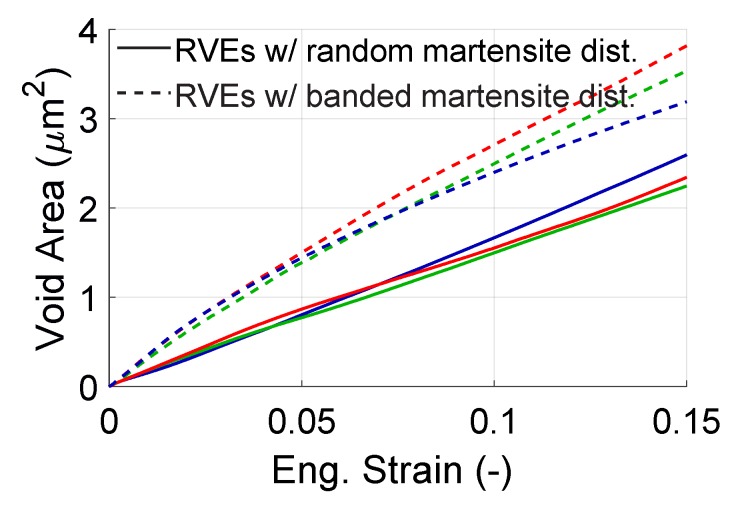
Evolution of total area of the voids formed by martensite cracks for 3 different orientation sets for random and banded distribution of martensite with total strain. Each color represents an orientation set.

**Figure 15:**
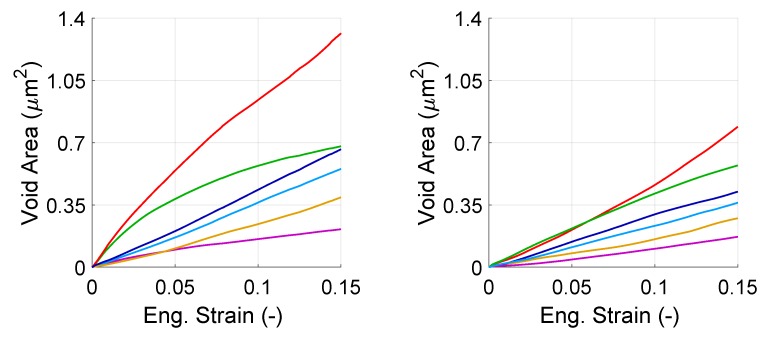
Evolution of the area of individual voids formed by martensite cracking in banded (**left**) and random (**right**) morphology with total strain. Each color represents one void.

In this part, we analyze the evolution of total area of the voids formed by martensite cracks as well as area of individual voids of that type. The area of this type of voids is defined as the area in the plane of the model, that is formed by opening of the faces of the martensite islands during deformation. In Figure 14, the damaged areas caused by the 6 voids were summed up which gives an overview on evolution of the voids in the structure. Figure 15 shows the evolution of damaged area that is induced by individual cracks. Since the results between different orientation sets were similar this figure was plotted only for one orientation per morphology.

From Figure 14, it is seen that for all orientation sets of banded morphology, growth of crack area occurred faster than for random morphology. This behavior can be expected since the martensite islands in a banded structure act as strong fibers and they carry more load compared to their counterpart in a random structure. This explains the faster growth for banded morphology. This was also verified by the $\rho_{\mathrm{SSD}}$ and $\rho_{\mathrm{GND}}$ distributions in Figure 9 and Figure 10 where it can be seen that the average dislocation density of martensite in banded morphology is slightly higher than that of random morphology.

In Figure 15, it is seen that for both morphologies all cracks open, but with different rates. This can be explained by the variation in the local stress state surrounding the crack. In this manner, it is similar to the evolution of individual voids. The trends of different cracks in random morphology looks more similar than in banded morphology. In the banded morphology, there is only one case that shows a saturating trend. The voids formed by martensite cracking grow in a more monotonic way—in the sense that growth trend does not change during the deformation—than the voids at the ferrite–martensite interfaces and in ferrite grains.

## 5. Summary and Conclusions

In this work, we have investigated and compared the effect of martensite morphology on damage evolution mechanisms of dual phase steels within a strain gradient enhanced crystal plasticity framework, which enables incorporation of plastic anisotropy as well as microstructural size effects. Two industrially relevant martensite morphologies (banded and random) are investigated by generated artificial 2D RVEs. To the RVEs, the most common damage mechanisms are: voids formed by martensite cracks, voids between ferrite and martensite, and voids in ferrite grains were introduced. After 15% tensile deformation it was found that:

- The morphology of the martensite phase in dual phase steels has a direct effect on the stress and plastic strain distribution among the phases.
- The size of the observed shear bands is strongly influenced by the average spacing between martensite islands.
- An accurate prediction of the local stress state is necessary to capture the evolution of individual voids.
- The heterogeneity of the plastic strain within an RVE results in significant GND densities which can be captured using a gradient enhanced crystal plasticity model.
- Local stress state around a void varies significantly from the applied average stress state and it evolves considerably during deformation.
- The change of stress state around a void during large plastic deformation may lead to closure of the void.
- Voids that are formed by cracking of martensite exhibit the highest growth rates, thus they are considered as the most severe damage mechanism.

## Figures and Tables

**Table 1:** Coefficients of interaction matrix $Q^{\left(\alpha \beta \right)}$ for body centered cubic (BCC)-Fe [51].

Self	Coplanar	Collinear	Orthogonal	Glissile	Sessile
0.009	0.009	0.72	0.05	0.09	0.06

**Table 2:** Chemical composition (wt %) of the DP600 steel (TATA Steel, Ijmuiden, The Netherlands) after which the representative volume elements (RVEs) are modeled.

C	Cr	Mn	Si	P & S
0.09	0.5	1.9	0.06	trace

**Table 3:** Material parameters for ferrite and martensite.

	Ferrite	Martensite
E (GPa)	212	212
ν (-)	0.3	0.3
$\tau_{0}$ (MPa)	40	250
*b* (mm)	$2.48\times 10^{-7}$	$2.48\times 10^{-7}$
$\rho_{0}$ (mm${}^{-2}$)	$7.50\times 10^{6}$	$1.34\times 10^{8}$
$\rho^{\infty }$ (mm${}^{-2}$)	$3.00\times 10^{8}$	$5.00\times 10^{8}$
$\gamma^{\infty }\left(-\right)$	0.4	0.3

## Nomenclature

*b*	Burgers vector length.
BCC	Body Centered Cubic crystal structure.
$C^{e}$	Elasticity tensor.
${\hat{D}}_{e}$	Elastic rate of deformation between the lattice and the deformed configurations, defined in the deformed configuration.
${\hat{D}}_{i}$	Inelastic rate of deformation between the reference and lattice configurations, defined in the deformed configuration.
D	Rate of deformation that is the symmetric part of the velocity gradient and a measure of the rate of strain between the reference and the deformed configurations, defined in the deformed configuration.
${\hat{F}}_{e}$	Deformation gradient that describes elastic stretching and lattice rotation of the plastically deformed material point at an intermediate configuration (#).
F	Deformation gradient that takes a line element of the material $dx_{0}$ from an undeformed, reference configuration to dx in the final deformed configuration as $dx=F\;\cdot \;dx_{0}$.
$F_{i}$	Deformation gradient that describes the inelastic deformation caused by glide of dislocations and takes a line element from the reference configuration to an intermediate (lattice) configuration.
FCC	Face Centered Cubic crystal structure.
${\dot{\gamma }}^{\left(\alpha \right)}$	Shear rate on slip system α.
$\gamma^{\infty }$	Phenomenological constant that controls the rate of saturation of the Statistically Stored Dislocation density.
G	Burgers tensor.
$\overset{□}{G}$	Plastically convected rate of the Burgers tensor.
GND	Geometrically Necessary Dislocations.
${\hat{L}}_{e}$	Velocity gradient that gives the gradient of the velocity field (between the lattice and deformed configuration) at the final deformed configuration.
${\hat{L}}_{i}$	Velocity gradient that gives the gradient of the velocity field (between the reference and lattice configuration) at the final deformed configuration.
L	Velocity gradient that gives the gradient of the velocity field (between the reference and deformed configuration) at the final deformed configuration.
$l^{\left(\alpha \right)}$	A lattice vector that is orthogonal to both s and m.
$m^{\left(\alpha \right)}$	Slip plane normal vector on slip system α given at the deformed configuration.
$m_{l}^{\left(\alpha \right)}$	Normal to the non-glide plane on slip system α observed in BCC crystals.
$m_{0}^{\left(\alpha \right)}$	Slip plane normal vector on slip system α given at the reference configuration.
μ	Shear modulus.
$\nabla_{0}$	Gradient operator in the reference configuration.
$\nabla_{\#}$	Gradient operator in the lattice configuration.
$P_{\mathrm{ns}}^{\left(\alpha \right)}$	Contribution of non-Schmid effects seen in BCC crystals to the total Schmid tensor.
$P_{s}^{\left(\alpha \right)}$	Schmid tensor on slip system α, i.e., $P_{s}^{\left(\alpha \right)}=s^{\left(\alpha \right)}⊗\;m^{\left(\alpha \right)}$
$\phi^{\left(\alpha \right)}$	Flow criterion for slip system α.
$Q^{\left(\alpha \beta \right)}$	Physically based interaction matrix relating the hardening of slip system α to the dislocation density on slip system β.
Δr	Scalar distance between the points used in the computation of shear gradients.
${\hat{R}}_{e}$	Rotation resulting from the elastic deformation between the lattice and the deformed configuration as well as rigid rotation.
R	Total rotation of a line element of the material from the reference configuration to the deformed configuration, calculated using Polar Decomposition.
r	Position vector that is used for the computation shear rate gradients.
$\rho_{\mathrm{GND}}^{\left(\alpha \right)}$	Density of Geometrically Necessary Dislocations on slip system α.
$\rho_{\mathrm{SSD}}^{\left(\alpha \right)}$	Density of Statistically Stored Dislocations on slip system α.
$\rho^{\left(\beta \right)}$	Total dislocation density of the slip system β.
$\rho_{\mathrm{SSD}}^{\infty }$	Phenomenological constant that controls the saturation level of Statistically Stored Dislocation density.
σ	Cauchy stress, defined in the deformed configuration.
$s^{\left(\alpha \right)}$	Slip direction vector on slip system α given at the deformed configuration.
$s_{0}^{\left(\alpha \right)}$	Slip direction vector on slip system α given at the reference configuration.
$\overset{▿}{\sigma }$	An objective rate of the Cauchy stress tensor, in Abaqus/Standard it is updated using the corotational formulation.
SSD	Statistically Stored Dislocations.

$\tau^{\left(\alpha \right)}$	Resolved shear stress on slip system α.
$\tau_{f}^{\left(\alpha \right)}$	Critical resolved shear stress on slip system α.
$\tau_{0}$	Strain independent lattice friction.
${\hat{W}}_{e}$	Spin resulting from the elastic deformation between the lattice and the deformed configurations and rigid rotation, defined in the deformed configuration.
${\hat{W}}_{i}$	Spin resulting from the inelastic deformation between the reference and lattice configurations, defined in the deformed configuration.
W	Spin tensor that is the skew-symmetric part of the velocity gradient and a measure of the rate of rotation between the reference and the deformed configurations, defined in the deformed configuration.
Y	Numerically calculated gradient of the shear rates.

## Appendix A. Large Deformation Implementation of Crystal Plasticity

Resulting from the multiplicative decomposition of the total deformation gradient into elastic and inelastic parts, the total velocity gradient L is calculated as

(A1) $$L=\dot{F}\;\cdot \;F^{-1}=\left({\dot{\hat{F}}}_{e}\;\cdot \;F_{i}+{\hat{F}}_{e}\;\cdot \;{\dot{F}}_{i}\right)\;\cdot \;F_{i}^{-1}\;\cdot \;{\hat{F}}_{e}^{-1}$$

(A2) $$L={\hat{L}}_{e}+{\hat{F}}_{e}\;\cdot \;L_{i}\;\cdot \;{\hat{F}}_{e}^{-1}={\hat{L}}_{e}+{\hat{L}}_{i}$$

The inelastic part of the total velocity gradient tensor is calculated by the sum of shear rates (${\dot{\gamma }}^{\left(\alpha \right)}$) of the slip systems as [32,33]

(A3) $$L_{i}=\sum_{\alpha }{\dot{\gamma }}^{\left(\alpha \right)}s_{0}^{\left(\alpha \right)}⊗\;m_{0}^{\left(\alpha \right)}\;,\;{\hat{L}}_{i}=\sum_{\alpha }{\dot{\gamma }}^{\left(\alpha \right)}s^{\left(\alpha \right)}⊗\;m^{\left(\alpha \right)}$$

where

(A4) $$s^{\left(\alpha \right)}={\hat{F}}_{e}\;\cdot \;s_{0}^{\left(\alpha \right)}\;,\;m^{\left(\alpha \right)}={\hat{F}}_{e}^{-T}\;\cdot \;m_{0}^{\left(\alpha \right)}$$

Furthermore by using the additive decomposition of the velocity gradient into rate of deformation (D) and spin tensor (W), from Equation (A2) one can obtain

(A5) $$L={\hat{D}}_{e}+{\hat{F}}_{e}\;\cdot \;D_{i}\;\cdot \;{\hat{F}}_{e}^{-1}+{\hat{W}}_{e}+{\hat{F}}_{e}\;\cdot \;W_{i}\;\cdot \;{\hat{F}}_{e}^{-1}$$

where

(A6a) $$\begin{array}{cc} D & ={\hat{D}}_{e}+{\hat{F}}_{e}\;\cdot \;D_{i}\;\cdot \;{\hat{F}}_{e}^{-1}={\hat{D}}_{e}+{\hat{D}}_{i} \end{array}$$

(A6b) $$\begin{array}{cc} W & ={\hat{W}}_{e}+{\hat{F}}_{e}\;\cdot \;W_{i}\;\cdot \;{\hat{F}}_{e}^{-1}={\hat{W}}_{e}+{\hat{W}}_{i} \end{array}$$

Here, a common simplification is introduced on ${\hat{F}}_{e}$ such that ${\hat{R}}_{e}\gg {\hat{U}}_{e}$ and ${\hat{U}}_{e}≅I$ since the elastic stretch of a metal is rather small. This implies that the length of unit vectors $s_{0}^{\left(\alpha \right)}$ and $m_{0}^{\left(\alpha \right)}$ do not change during deformation but only rotate with ${\hat{R}}_{e}$ [16,62,63,64] which simplifies Equation (A6) to

(A7a) $$\begin{array}{cc} D & ≅{\hat{D}}_{e}+{\hat{R}}_{e}\;\cdot \;D_{i}\;\cdot \;{\hat{R}}_{e}^{-1}≅{\hat{D}}_{e}+{\hat{D}}_{i} \end{array}$$

(A7b) $$\begin{array}{cc} W & ≅{\hat{W}}_{e}+{\hat{R}}_{e}\;\cdot \;W_{i}\;\cdot \;{\hat{R}}_{e}^{-1}≅{\hat{W}}_{e}+{\hat{W}}_{i} \end{array}$$

In a finite element based time integration scheme the incremental rotation (ΔR) can be calculated from the exponential form of the known spin tensor (W) as [65]

(A8) $$\Delta R=\exp\left(W\right)≅\left(I+\frac{1}{2}W\right)\;\cdot \;{\left(I-\frac{1}{2}W\right)}^{-1}$$

and the total rotation at the end of the time step (t+1) can be obtained from

(A9) $$R^{t+1}=\Delta R\;\cdot \;R^{t}$$

With this, and considering that the total spin during the increment is known, now the total elastic rotation of the slip systems can be found using [66]:

(A10) $${\hat{R}}_{e}^{t+1}=\exp\left({\hat{W}}_{e}\right)\;\cdot \;{\hat{R}}_{e}^{t}=\exp\left(W-{\hat{W}}_{i}\right)\;\cdot \;{\hat{R}}_{e}^{t}$$

## Appendix B. Large Deformation Implementation of Strain Gradient Enhancement

We start by defining the Burgers tensor G, which characterizes the closure failure of an initially closed referential circuit as

(A11) $$G=F_{i}\;\cdot \;\mathrm{Curl}\;F_{i}$$

and plastically convected rate of G as [67]:

(A12) $$\overset{□}{G}=\sum_{\alpha }\left(\nabla_{\#}\dot{\gamma }\times m^{\left(\alpha \right)}\right)⊗s^{\left(\alpha \right)}$$

where $\nabla_{\#}\dot{\gamma }$ can be viewed as the gradient of $\dot{\gamma }$ in the lattice configuration (#), in which the lattice is undistorted and in intermediate configuration [54]. We can define a unit lattice vector $l^{\left(\alpha \right)}$ as $l^{\left(\alpha \right)}=m^{\left(\alpha \right)}\times s^{\left(\alpha \right)}$. Then, by using the orthogonality of $\left(\nabla_{\#}\dot{\gamma }\times m^{\left(\alpha \right)}\right)$ to $m^{\left(\alpha \right)}$, $\overset{□}{G}$ can be expanded as

(A13) $$\overset{□}{G}=\sum_{\alpha }\left(l^{\left(\alpha \right)}\;\cdot \;\nabla_{\#}{\dot{\gamma }}^{\left(\alpha \right)}s^{\left(\alpha \right)}⊗s^{\left(\alpha \right)}-s^{\left(\alpha \right)}\;\cdot \;\nabla_{\#}{\dot{\gamma }}^{\left(\alpha \right)}l^{\left(\alpha \right)}⊗s^{\left(\alpha \right)}\right)$$

By definition, both the direction of Burgers vector and dislocation line direction is equal and parallel to $s^{\left(\alpha \right)}$ for screw dislocations ($s^{\left(\alpha \right)}=l^{\left(\alpha \right)}$) while for edge dislocations the direction of Burgers vector is $s^{\left(\alpha \right)}$ and dislocation line direction is $l^{\left(\alpha \right)}$ and they are perpendicular to each other ($s^{\left(\alpha \right)}⊢l^{\left(\alpha \right)}$). Then the rate of screw (⊙) and edge (⊢) dislocation densities due to lattice incompatibility in a slip system α can be calculated from the dislocation line and the gradient of slip rate vector as:

(A14a) $$\begin{array}{c} {\dot{\rho }}_{⊙,\mathrm{GND}}^{\left(\alpha \right)}=l^{\left(\alpha \right)}\;\cdot \;\nabla_{\#}{\dot{\gamma }}^{\left(\alpha \right)}, \end{array}$$

(A14b) $$\begin{array}{c} {\dot{\rho }}_{⊢,\mathrm{GND}}^{\left(\alpha \right)}=-s^{\left(\alpha \right)}\;\cdot \;\nabla_{\#}{\dot{\gamma }}^{\left(\alpha \right)}, \end{array}$$

However, this calculation has to be done in the lattice configuration. On the other hand, the use of USDFLD—Abaqus user subroutine for field calculations—algorthmically limits the calculations to be done in either reference or deformed configuration. In order to push forward or pull back, one can employ the identities [55]

(A15) $$\nabla_{\#}{\dot{\gamma }}^{\left(\alpha \right)}=F_{i}^{-T}\;\cdot \;\nabla_{0}{\dot{\gamma }}^{\left(\alpha \right)}=F_{e}^{T}\;\cdot \;\nabla {\dot{\gamma }}^{\left(\alpha \right)}$$

Here our choice was to push forward from lattice configuration # to reference configuration and yield the GND evolution equations as [54]

(A16a) $$\begin{array}{c} {\dot{\rho }}_{⊙,\mathrm{GND}}^{\left(\alpha \right)}=l^{\left(\alpha \right)}\;\cdot \;F_{i}^{-T}\;\cdot \;\nabla_{0}{\dot{\gamma }}^{\left(\alpha \right)}=l_{0}^{\left(\alpha \right)}\;\cdot \;\nabla_{0}{\dot{\gamma }}^{\left(\alpha \right)} \end{array}$$

(A16b) $$\begin{array}{c} {\dot{\rho }}_{⊢,\mathrm{GND}}^{\left(\alpha \right)}=-s^{\left(\alpha \right)}\;\cdot \;F_{i}^{-T}\;\cdot \;\nabla_{0}{\dot{\gamma }}^{\left(\alpha \right)}=-s_{0}^{\left(\alpha \right)}\;\cdot \;\nabla_{0}{\dot{\gamma }}^{\left(\alpha \right)} \end{array}$$
